# Supplementary material for: Mapping global urban land for the 21st century with data-driven simulations and Shared Socioeconomic Pathways
Source: Nat Commun. 2020 May 8;11:2302. doi: 10.1038/s41467-020-15788-7 (PMC7210308; doi:10.1038/s41467-020-15788-7)
Supplement: Supplementary file 1 — Supplementary Information [file 41467_2020_15788_MOESM1_ESM.pdf]

Supplementary Information:

Mapping Global Urban Land for the 21st Century with Data-Driven Simulations and Shared Socioeconomic Pathways

Gao & O'Neill

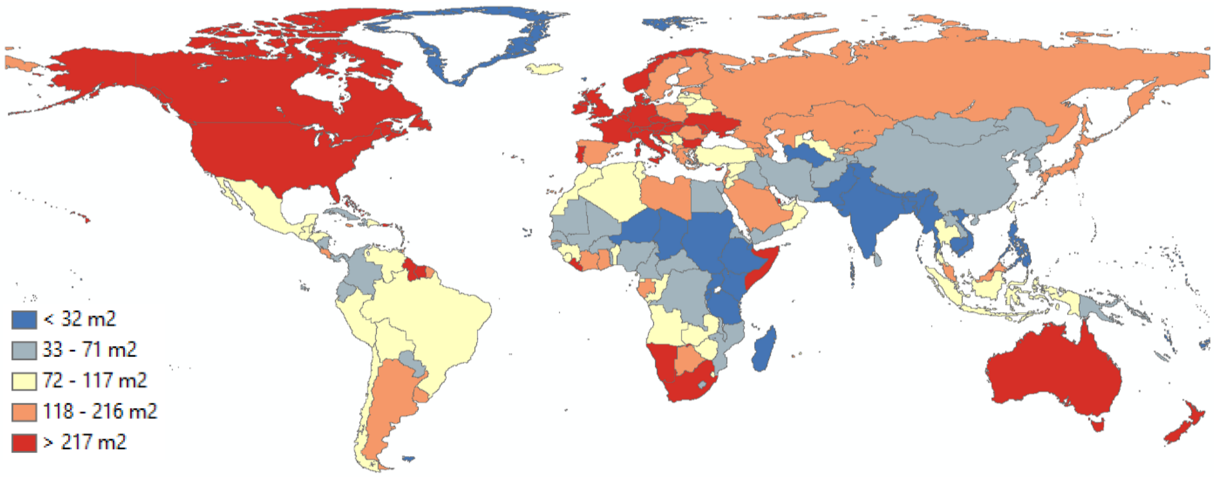

Supplementary Figure 1. Global quintile map of per capita urban land area (m2) in 2000. (Source data are provided as a Source Data file.)

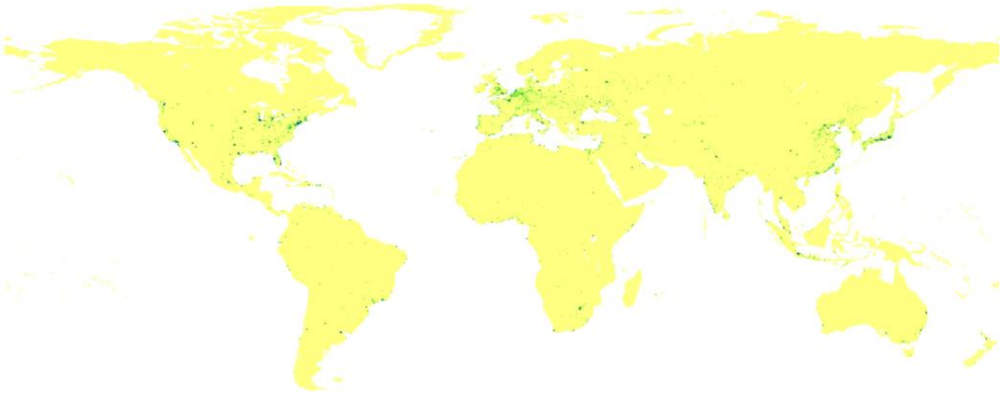

(a)

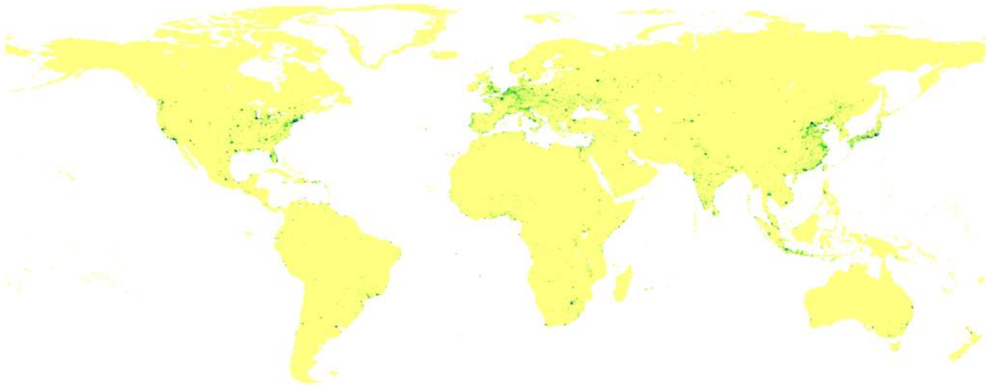

(b)

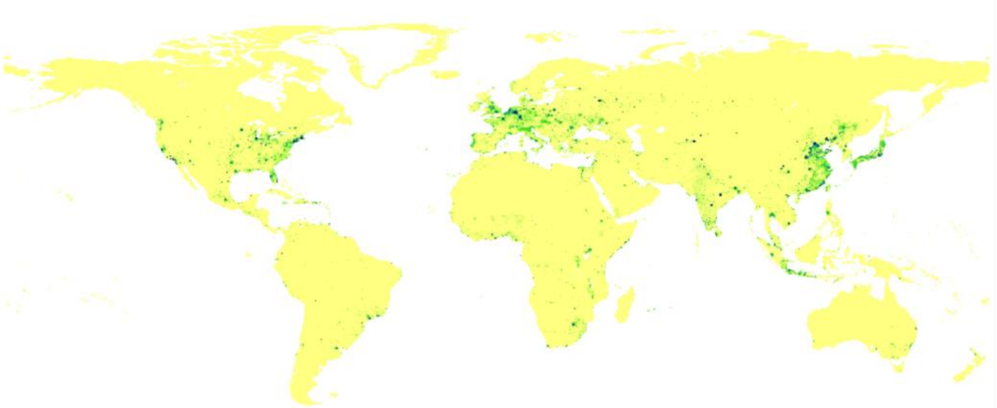

(c)

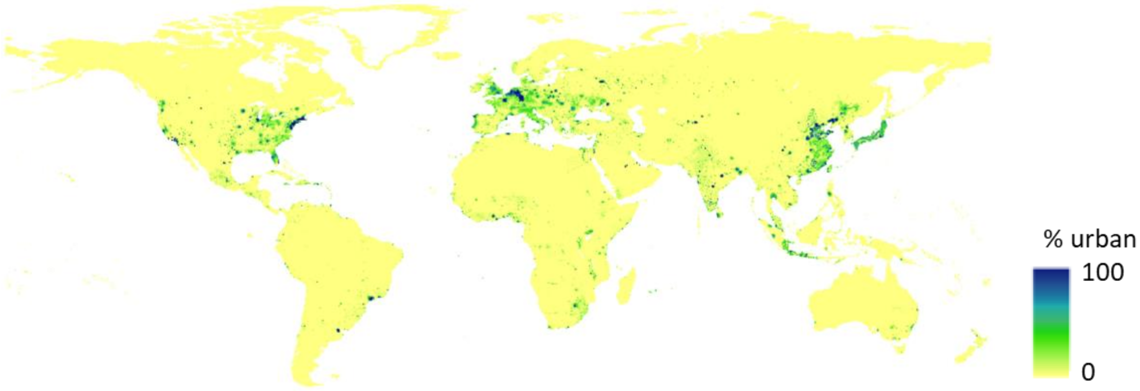

(d)

Supplementary Figure 2. Global urban land maps: (a) base year 2000, (b) sustainability (SSP 1) 2100, (c) middle of the road (SSP 2) 2100, and (d) fossil-fueled development (SSP 5) 2100.

Supplementary Table 1. Urban land expansion of world regions over the 21st century. When comparing total urban areas across regions, readers should bear in mind that the regions have different land areas.

	Base Year 2000		Middle of Road Scenario: 2000-2100 Change						
	total urban land (km2)	per capita urban land (m2)	per capita urban land 2100 (m2)	total urban land change (km2)	urban land change %	total pop change %	urban pop change %	GDP change %	2100/2000 urban land ratio (range across scenarios)
North America	133,611	424	686	218,560	164	63	99	281	1.4 - 4.6
South & Central America	51,373	98	203	85,482	166	28	58	947	1.5 - 4.9
Europe	144,173	248	723	275,285	191	0	30	321	1.6 - 5.0
Russia	21,533	147	490	38,492	179	-16	6	639	1.5 - 4.7
North Africa & Middle East	33,736	86	143	74,747	222	94	181	1,457	1.7 - 5.3
Africa	46,067	69	100	193,215	419	260	744	10,340	2.5 - 8.0
East & Central Asia	113,944	66	397	450,839	396	-18	59	1,001	2.2 - 7.5
South & Southeast Asia	55,032	32	125	253,963	461	42	252	3,450	2.7 - 8.4
Oceania	11,272	382	460	17,815	158	114	164	727	1.4 - 4.6
Global Total	610,742	100	246	1,608,399	263	47	152	1,035	1.8 - 5.9

Supplementary Table 2. Urban expansion style evolution of example countries shown by historical data.

Nation	1990-2000	2000-2010
Nigeria	3	2
China	3	2
India	3	2
Malaysia	2	1
South Africa	2	1
Ireland	2	1

(3 – rapidly urbanizing; 2 – steadily urbanizing; 1 – urbanized)

Supplementary Table 3. Urban expansion style evolution projected for China.

	2010	2020	2030	2040	2050	2060	2070	2080	2090	2100
SSP 1	2	2	1	1	1	1	1	1	1	1
SSP 2	2	2	2	1	1	1	1	1	1	1
SSP 3	2	2	2	2	1	1	1	1	1	1
SSP 4	2	2	1	1	1	1	1	1	1	1
SSP 5	2	2	1	1	1	1	1	1	1	1

(2 – steadily urbanizing; 1 – urbanized)

Supplementary Table 4. Number of global countries following different urbanization styles in different scenarios.

	base year	SSP 2		SSP 5		SSP 1	
	2000	2050	2100	2050	2100	2050	2100
urbanized	113	179	208	205	214	188	214
steadily urbanizing	95	52	23	8	0	26	0
rapidly urbanizing	23	0	0	18	17	17	17

Supplementary Table 5. Urban land expansion of sample countries over the 21st century.

	Base Year 2000		Middle of Road Scenario: 2000-2100 Change						
	total urban land (km2)	per capita urban land (m2)	per capita urban land 2100 (m2)	total urban land change (km2)	urban land change %	total pop change %	urban pop change %	GDP change %	2100/2000 urban land ratio (range across scenarios)
United States of America	123,957	436	713	202,970	164	61	97	268	1.4 - 4.6
Brazil	17,766	102	237	26,912	151	8	26	648	1.3 - 4.5
Germany	24,220	290	1,048	45,514	188	-20	2	182	1.5 - 4.8
United Kingdom	12,892	219	401	21,064	163	44	71	397	1.3 - 4.4
Russia	21,533	147	490	38,492	179	-16	6	639	1.5 - 4.7
Ethiopia	1,086	16	63	10,993	1,012	189	1,096	23,326	5.7 - 17.1
South Africa	11,148	249	630	25,700	231	30	98	1,004	1.7 - 5.3
China	73,492	57	552	350,290	477	-40	35	1,658	2.4 - 8.6
India	21,631	21	97	134,480	622	54	313	4,535	3.3 - 10.2

Supplementary Table 6. Comparing 2000-2100 urban land expansion (ULE) % and total population change (TPC) % for world regions across scenarios.

	SSP1		SSP2		SSP3		SSP4		SSP5	
	ULE%	TPC%	ULE%	TPC%	ULE%	TPC%	ULE%	TPC%	ULE%	TPC%
North America	42	65	164	63	47	-8	163	29	360	154
South & Central America	48	-7	166	28	58	106	166	8	386	-14
Europe	61	-3	191	0	85	-32	192	-23	404	40
Russia	48	-37	179	-16	74	2	171	-40	373	-31
North Africa & Middle East	67	42	222	94	104	206	206	116	427	44
Africa	155	154	419	260	238	438	341	419	701	147
East & Central Asia	121	-35	396	-18	214	15	326	-18	652	-34
South & Southeast Asia	175	4	461	42	300	124	406	14	741	2
Oceania	40	96	158	114	46	60	155	103	358	192
Global Total	84	13	263	47	129	107	238	51	491	21
