# Supplementary material for: Mapping global urban land for the 21st century with data-driven simulations and Shared Socioeconomic Pathways
Source: Nat Commun. 2020 May 8;11:2302. doi: 10.1038/s41467-020-15788-7 (PMC7210308; doi:10.1038/s41467-020-15788-7)
Supplement: Supplementary file 3 — Reporting Summary [file 41467_2020_15788_MOESM3_ESM.pdf]

nature research

Corresponding author(s): Jing Gao

Last updated by author(s): Oct 10, 2019

Reporting Summary

Nature Research wishes to improve the reproducibility of the work that we publish. This form provides structure for consistency and transparency in reporting. For further information on Nature Research policies, see Authors & Referees and the Editorial Policy Checklist.

Statistics

For all statistical analyses, confirm that the following items are present in the figure legend, table legend, main text, or Methods section.

n/a	Confirmed	
☒	☐	The exact sample size (*n*) for each experimental group/condition, given as a discrete number and unit of measurement
☒	☐	A statement on whether measurements were taken from distinct samples or whether the same sample was measured repeatedly
☒	☐	The statistical test(s) used AND whether they are one- or two-sided<br>*Only common tests should be described solely by name; describe more complex techniques in the Methods section.*
☐	☒	A description of all covariates tested
☐	☒	A description of any assumptions or corrections, such as tests of normality and adjustment for multiple comparisons
☐	☒	A full description of the statistical parameters including central tendency (e.g. means) or other basic estimates (e.g. regression coefficient) AND variation (e.g. standard deviation) or associated estimates of uncertainty (e.g. confidence intervals)
☒	☐	For null hypothesis testing, the test statistic (e.g. *F*, *t*, *r*) with confidence intervals, effect sizes, degrees of freedom and *P* value noted<br>*Give P values as exact values whenever suitable.*
☒	☐	For Bayesian analysis, information on the choice of priors and Markov chain Monte Carlo settings
☐	☒	For hierarchical and complex designs, identification of the appropriate level for tests and full reporting of outcomes
☐	☒	Estimates of effect sizes (e.g. Cohen's *d*, Pearson's *r*), indicating how they were calculated

Our web collection on statistics for biologists contains articles on many of the points above.

Software and code

Policy information about availability of computer code

Data collection	This work used existing, publicly-accessible datasets.
Data analysis	This work used standard implementations of analysis methods from R-Studio and ArcGIS.

For manuscripts utilizing custom algorithms or software that are central to the research but not yet described in published literature, software must be made available to editors/reviewers. We strongly encourage code deposition in a community repository (e.g. GitHub). See the Nature Research guidelines for submitting code & software for further information.

Data

Policy information about availability of data

All manuscripts must include a data availability statement. This statement should provide the following information, where applicable:

- Accession codes, unique identifiers, or web links for publicly available datasets
- A list of figures that have associated raw data
- A description of any restrictions on data availability

Datasets produced by this work (i.e. national total amounts of urban land, and time series maps of urban land, under urbanization scenarios consistent with the SSPs) are publicly downloadable at dataverse.harvard.edu/dataverses/geospatial_human_dimensions_data . The raw data for all figures displaying contents other than these projections are provided in the Source Data file.

Field-specific reporting

Please select the one below that is the best fit for your research. If you are not sure, read the appropriate sections before making your selection.

☐ Life sciences ☐ Behavioural & social sciences ☒ Ecological, evolutionary & environmental sciences

For a reference copy of the document with all sections, see nature.com/documents/nr-reporting-summary-flat.pdf

Ecological, evolutionary & environmental sciences study design

All studies must disclose on these points even when the disclosure is negative.

Study description	This study analyzed publicly available datasets on global urbanization and its driving forces to identify empirical relationships among variables, and used such relationships to model future urbanization under alternative socioeconomic scenarios.
Research sample	The study used complete global datasets including all countries and all land areas. No sampling was done.
Sampling strategy	The study used complete global datasets including all countries and all land areas. No sampling was done.
Data collection	This work used existing, publicly-accessible datasets. No data was collected by the authors.
Timing and spatial scale	The existing datasets used in this study cover the time horizon of 1980-2010 at decadal intervals, and offer the spatial scales of national aggregates and 1/8-degree grid cells.
Data exclusions	No data were excluded.
Reproducibility	Our experiments are all computational only. Everything is reproducible.
Randomization	Randomization was not relevant, because no comparative experiment was involved.
Blinding	Blinding was not relevant, because no data collection nor comparative experiment was involved.

Did the study involve field work? ☐ Yes ☒ No

Reporting for specific materials, systems and methods

We require information from authors about some types of materials, experimental systems and methods used in many studies. Here, indicate whether each material, system or method listed is relevant to your study. If you are not sure if a list item applies to your research, read the appropriate section before selecting a response.

Materials & experimental systems

n/a	Involved in the study	
☒	☐	Antibodies
☒	☐	Eukaryotic cell lines
☒	☐	Palaeontology
☒	☐	Animals and other organisms
☒	☐	Human research participants
☒	☐	Clinical data

Methods

n/a	Involved in the study	
☒	☐	ChIP-seq
☒	☐	Flow cytometry
☒	☐	MRI-based neuroimaging
